# Comparing internal jugular vein and subclavian vein for central venous insertion of implantable ports in cancer chemotherapy: a meta-analysis of RCTs

**DOI:** 10.3389/fonc.2025.1566757

**Published:** 2025-05-26

**Authors:** Qianqian Li, Xuemei Peng, Xia Leng, Suping Xiao, Meilan Min, Shufang Wan, Jianyun Wen

**Affiliations:** ^1^ Specialized Medical Department, Jiangxi Cancer Hospital & Institute (The Second Affiliated Hospital of Nanchang Medical College), Nanchang, China; ^2^ Department of Breast Oncology Surgery, Jiangxi Cancer Hospital & Institute (The Second Affiliated Hospital of Nanchang Medical College), Nanchang, China; ^3^ Department of Head and Neck Oncology Radiation Therapy, Jiangxi Cancer Hospital & Institute (The Second Affiliated Hospital of Nanchang Medical College), Nanchang, China

**Keywords:** internal jugular vein, subclavian vein, implantable port, complication, meta-analysis, randomized controlled trials

## Abstract

**Background:**

Central venous access for cancer chemotherapy is crucial for patients undergoing long-term treatment. The internal jugular vein (IJV) and subclavian vein (SCV) are commonly used for implantable port insertion, though the optimal choice remains debated. This meta-analysis aims to compare the safety and effectiveness of IJV and SCV for central venous implantable port insertion in cancer chemotherapy patients based on randomized controlled trials (RCTs).

**Methods:**

We systematically reviewed RCTs comparing IJV and SCV for implantable port insertion. The primary endpoint was complication, while secondary endpoints included procedure failure rate, procedure duration, patient satisfaction, and pain perception.

**Results:**

A total of 7 studies based on 6 RCTs met the inclusion criteria. The baseline characteristics (age, sex, port side, and duration of implant) of patients in both groups were comparable. According to patient-reported outcomes, the SCV group experienced higher rates of total complications (risk ratio [RR]: 0.52 [0.29, 0.93], P = 0.03, *I* ^2^ = 71%), catheter misplacement (RR: 0.51 [0.27, 0.96], P = 0.04, *I* ^2^ = 35%), and port/catheter-related bloodstream infections (RR: 0.37 [0.17, 0.81], P = 0.01, *I* ^2^ = 0%). Similarly, according to catheter days, the SCV group achieved higher rates of total complications (RR: 0.48 [0.35, 0.67], P < 0.0001, *I* ^2^ = 29%) and port/catheter-related bloodstream infections (PRBIs) (RR: 0.32 [0.14, 0.72], P = 0.006, *I* ^2^ = 0%). Pain perception (mean difference [MD]: -1.60 [-1.93, -1.27], P < 0.00001) was also worse in the SCV group. However, the duration of the procedure (MD: 11.55 [0.57, 22.54] minutes, P = 0.04, *I* ^2^ = 97%) was longer in the IJV group. The procedural failure rate was comparable between the two groups.

**Conclusions:**

For cancer chemotherapy patients, the IJV appears to be a safer and less painful alternative to the SCV for central venous port insertion.

**Systematic review registration:**

https://www.crd.york.ac.uk/prospero/, identifier CRD42025641904.

## Introduction

For cancer patients receiving chemotherapy, central venous access is critical, providing a reliable route for drug administration and blood sampling (1). Among the various methods, implantable ports (IPs) are preferred due to their lower complication rates and longer patency compared to other central venous devices (2). The internal jugular vein (IJV) and subclavian vein (SCV) are frequently used for IP insertion, but the optimal choice remains a subject of debate, as each approach presents distinct advantages and challenges (3). The IJV is often favored due to its more straightforward anatomical path and direct connection to the superior vena cava, which reduces the risk of catheter malposition. However, it is linked to an increased risk of complications, such as pneumothorax, particularly in patients with anatomical variations (4). The SCV, while deeper and more challenging to access, is less prone to displacement. However, it has been associated with a higher incidence of thrombosis and long-term catheter dysfunction (5).

Despite the widespread use of both venous sites, there remains a lack of high-quality, evidence-based studies comparing the clinical outcomes of IJV versus SCV for IP insertion. Several studies have explored various complications associated with these two approaches, including infection rates, thrombosis, and catheter migration, but the findings remain inconsistent. Mansfield et al. reported a lower incidence of pneumothorax with IJV access, while Rixecker et al. found a reduced infection rate with SCV access, likely due to its deeper anatomical location (6, 7). Additionally, Kaul et al. observed a higher incidence of thrombosis with SCV access, which complicates chemotherapy administration (8). Long-term complications, such as port migration and dysfunction, also differ between the two approaches. Becker et al. noted a higher risk of port migration with IJV insertion, which may require reintervention (9), whereas Adrian et al. found that SCV access is more prone to catheter occlusion over time, thereby reducing the port’s functionality for chemotherapy (10).

This meta-analysis aims to address the gap in current literature by comparing the safety and efficacy of IJV versus SCV for IP insertion in cancer patients. We focus on complications such as thrombosis and infection, along with clinical outcomes like pain perception. By synthesizing data from high-quality randomized controlled trials (RCTs), this study will provide a comprehensive evaluation of these two central venous access methods, offering evidence-based guidance for clinical practice.

## Materials and methods

### Search strategy

A comprehensive search was conducted across the Web of Science, EMBASE, Cochrane Library, PubMed, ScienceDirect, and Scopus databases to identify RCTs comparing IJV and SCV for central venous implantable port insertion in cancer chemotherapy, up to January 2, 2025. The MeSH terms used in the search included “Internal Jugular Vein”, “Subclavian Vein”, and “Randomized”. Furthermore, eligible studies were identified by screening the reference lists of the included articles. The complete search strategies for each database, including exact search strings, date ranges, and language filters, are presented in **Supplementary Table S1**.

### Selection criteria

The inclusion criteria were set as follows: 1. RCTs; 2. Studies involving cancer patients receiving implantable ports and chemotherapy; 3. Direct comparison between IJC and SCV; 4. Includes the following outcomes: primary outcomes (complications), secondary outcomes (characteristics, data of procedures, etc).

Articles were excluded if they lacked primary data, or were meta-analyses, conference abstracts, case reports, or reviews.

### Data extraction

Data were extracted independently by two investigators, including study characteristics (country, duration, etc.), patient characteristics (e.g. sex, age), safety (e.g. total and individual complications), and procedural data (e.g. failure rates, procedure duration). Any discrepancies were resolved through data re-evaluation.

### Outcome assessments

Complications were evaluated based on patient count or catheter days. Pain perception was measured using the Faces Pain Scale-Revised (FPS-R), which ranges from 0 to 10, with higher scores indicating greater levels of perceived pain (the minimal clinically important difference [MCID] was 1.0) (11).

### Quality assessment for included studies

The RCTs were assessed for quality using the Cochrane Risk of Bias Tool and the Jadad scale, with a maximum score of 7 points. A score of 4 or above indicates high quality (12, 13). The overall quality of evidence was evaluated using the GRADE approach (14).

### Statistical analysis

Statistical analyses were performed using Stata 14.0 and RevMan 5.3 software. I² > 50% or P < 0.1 indicating substantial heterogeneity, in line with current meta-analytic guidelines (15). A random-effects model was applied in cases of high heterogeneity, while a fixed-effects model was used when heterogeneity was low. For dichotomous data, pooled risk ratios (RR) were calculated, whereas for continuous data, mean differences (MD) were used. In addition, publication bias was evaluated using funnel plot asymmetry test. A p-value of less than 0.05 was considered statistically significant for all tests.

## Results

### Search results

**Figure 1:**
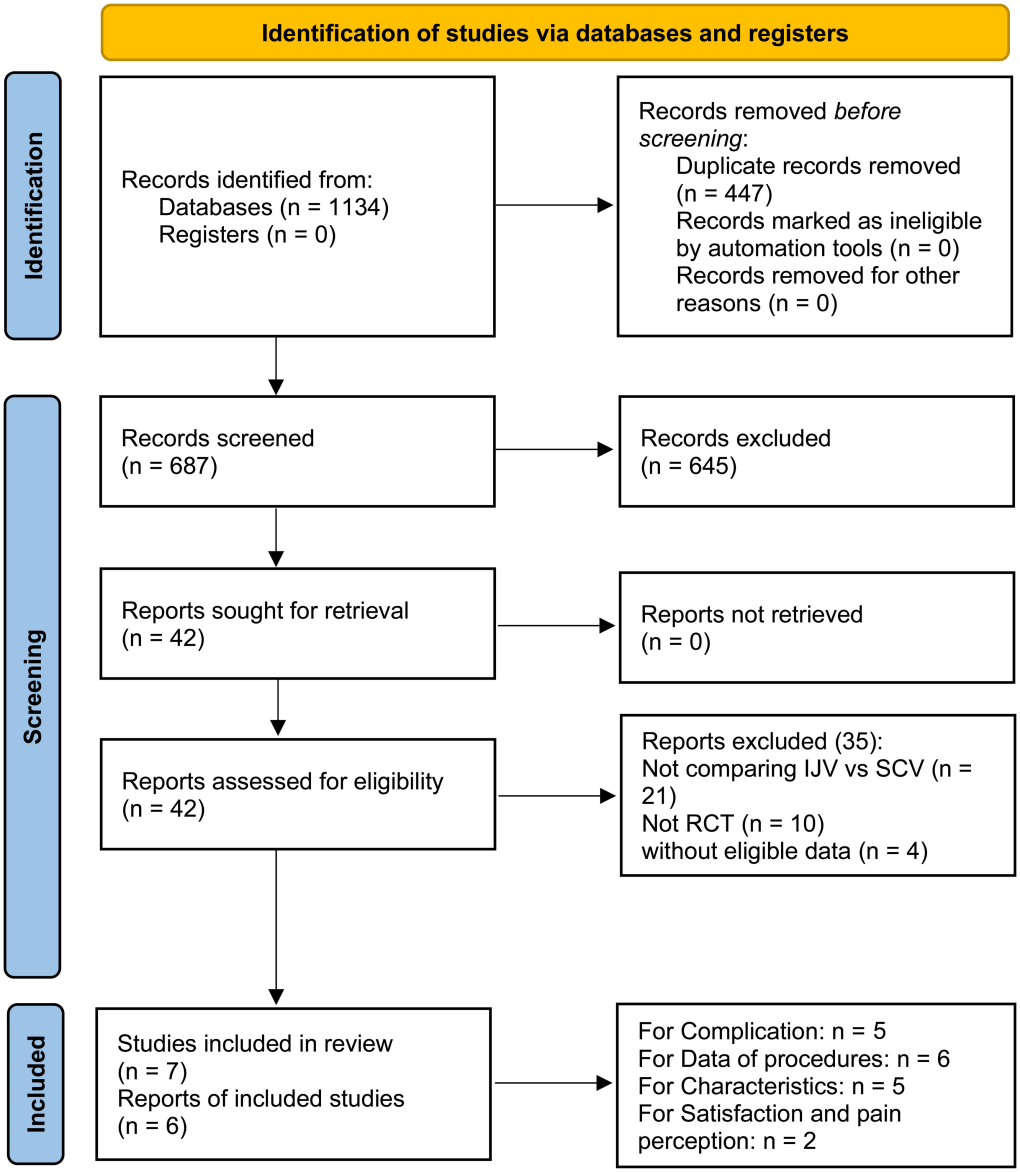
Flow chart. After an initial screening of 687 studies, 7 papers derived from 6 RCTs were included in the analysis.

**Figure 2:**
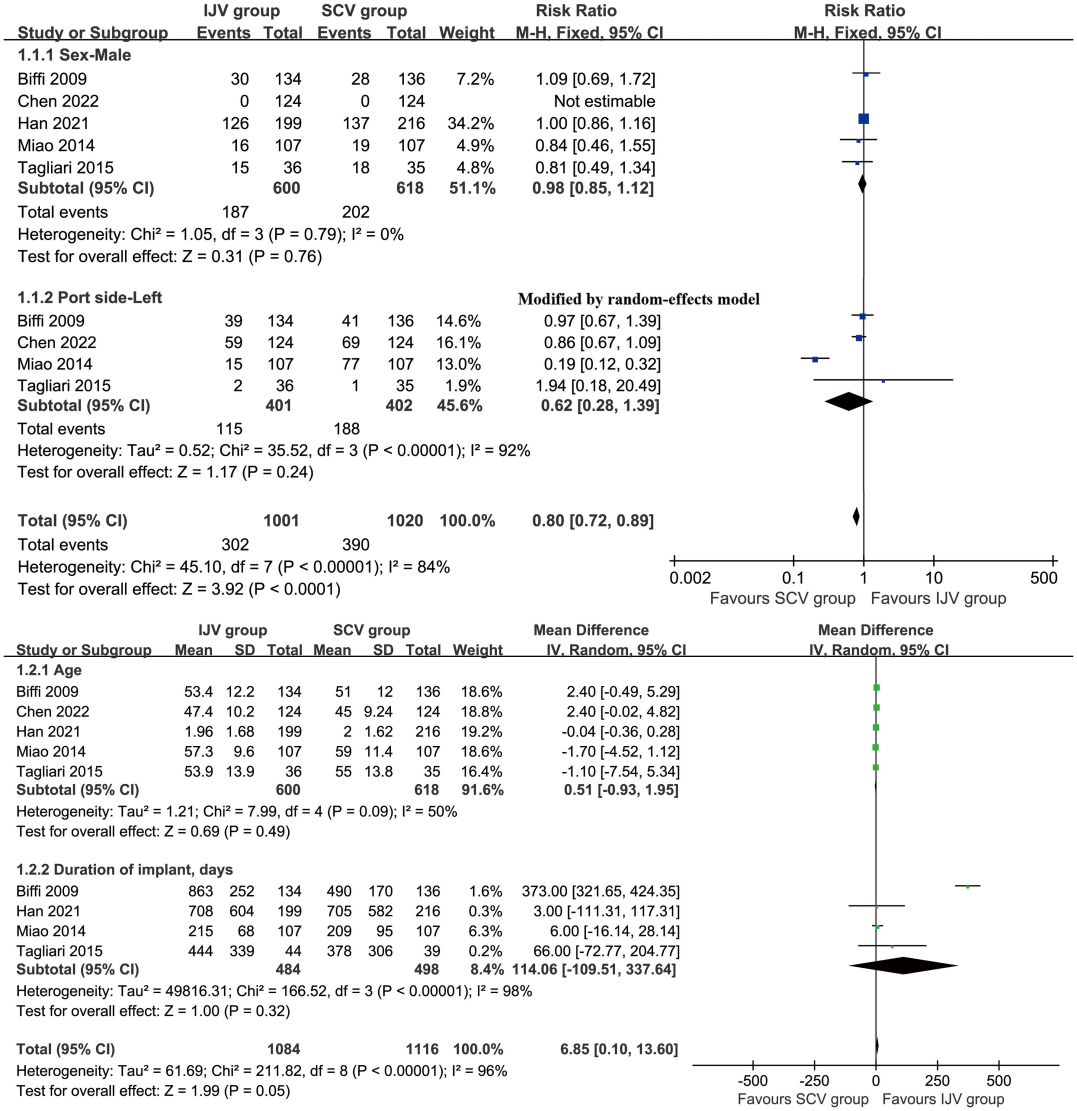
Forest plots of baseline characteristics associated with IJV versus SCV. Baseline age, sex, port side, and implant duration were comparable across both group.

After an initial screening of 687 studies, 7 papers derived from 6 RCTs were included in the analysis (IJV group: 644 patients; SCV group: 657 patients) (**Figure 1**) (16–22). The baseline characteristics are summarized in **Table 1**. Of the studies, three were conducted in Asia, two in South America, and one in Europe. When comparing baseline characteristics, age, sex, port side, and implant duration were comparable across both groups (**Figure 2**). Each of the studies was considered to be of high quality (**Supplementary Figure S1**, **Supplementary Table S2**). Additionally, based on the GRADE system, all results were rated as moderate to high quality (**Supplementary Table S3**).

**Table 1:** Baseline characteristics of the included randomized controlled trials.

Study	Country	Period (year)	Groups	Number of patients	Sex (M/F)	Age (Mean, year)	The puncture method	Quality
Biffi 2009 (15), Biffi 2014 (16)	Italy	2003.07-2006.12	IJV	134	30/104	53.4	Percutaneous landmark	6
			SCV	136	28/108	50.5	Ultrasonic	
Chen 2022 (17)	China	2020.08-2021.06	IJV	124	0/124	47.38	Ultrasonic	6
			SCV	124	0/124	45.05	Ultrasonic	
Han 2021 (18)	China	2015.04-2018.01	IJV	199	126/73	1.96	Ultrasonic	6
			SCV	216	137/79	1.96	Percutaneous landmark	
Miao 2014 (19)	China	2009.01-2013.06	IJV	107	16/91	57.3	Ultrasonic	5
			SCV	107	19/88	58.9	Percutaneous landmark	
Rodrigo 2012 (20)	Brazil	2004.01-2006.04	IJV	44	–	–	Percutaneous landmark	5
			SCV	39	–	–	Percutaneous landmark	
Tagliari 2015 (21)	Brazil	2014.08-2015.03	IJV	36	15/21	53.86	Percutaneous landmark	6
			SCV	35	18/17	54.86	Percutaneous landmark	

IJV, Internal Jugular Vein; M/F, Male/Female; SCV, Subclavian Vein.

### Complications assessed according to patients

**Figure 3:**
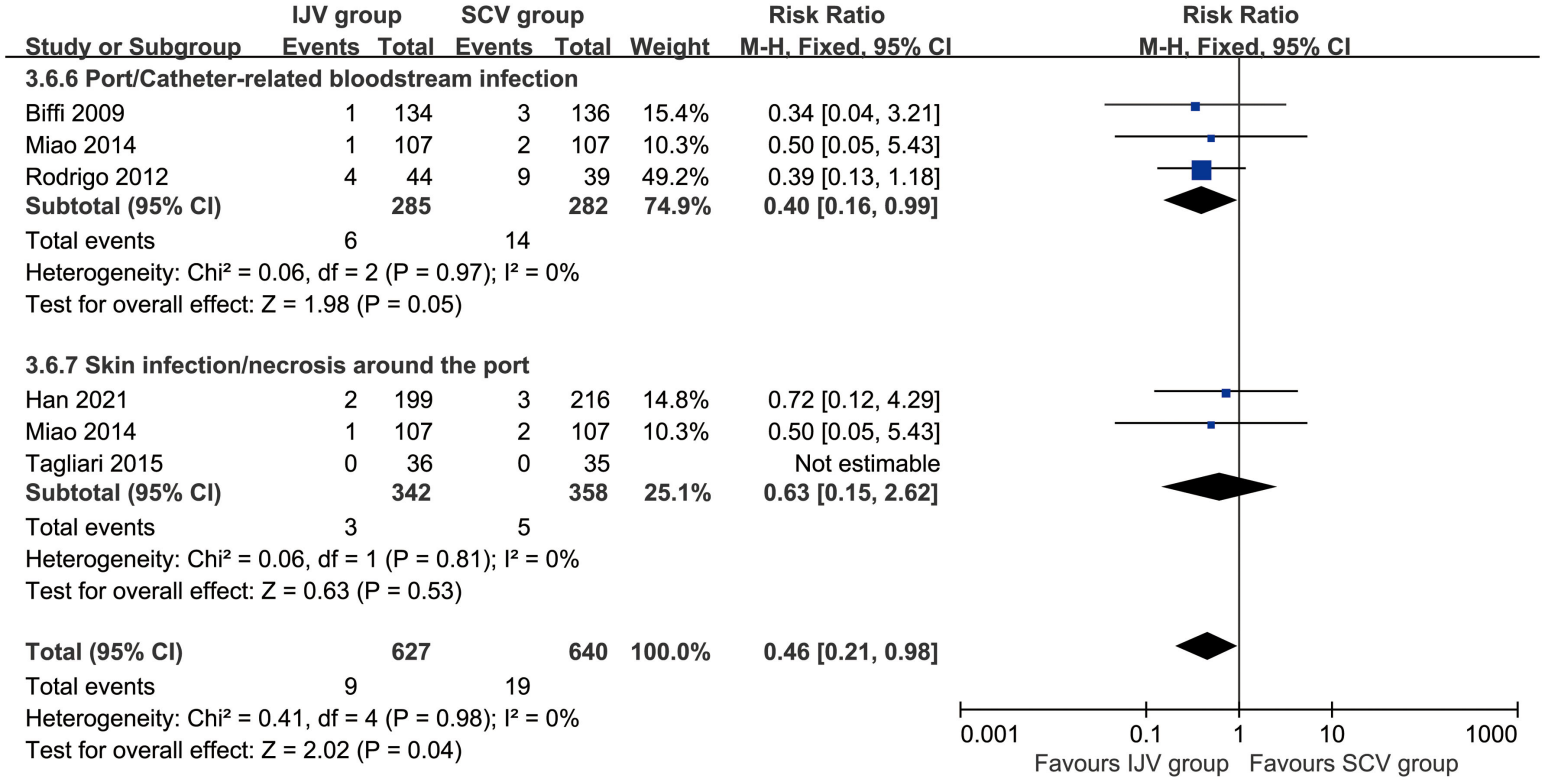
Forest plots of infections associated with IJV versus SCV. According to patient data, the SCV group exhibited higher rates of total complications, catheter misplacements, and port/catheter-related bloodstream infections.

According to patient data, the SCV group exhibited higher rates of total complications (RR: 0.52 [0.29, 0.93], P = 0.03, *I* ^2^ = 71%), catheter misplacements (RR: 0.51 [0.27, 0.96], P = 0.04, *I* ^2^ = 35%), and port/catheter-related bloodstream infections (PRBIs) (RR: 0.37 [0.17, 0.81], P = 0.01, *I* ^2^ = 0%) (**Figure 3**). The incidence of venous thrombosis, catheter occlusion, fibrin sleeve, port removal, catheter fracture, inadvertent artery puncture, subcutaneous hematoma, skin infection/necrosis around the port, pneumothorax, and infiltration/extravasation were comparable between the two groups (**Table 2**, **Supplementary Figure S2**).

**Table 2:** Complications assessed according to patients.

Complications assessed according to patients	Studies involved	IJV		SCV		Risk ratio [95% CI]	*I^2^*	P-value
		Event/total	%	Event/total	%			
**Total**	5	60/608	9.87%	110/622	17.68%	0.52 [0.29, 0.93]	71%	0.03
**Complications**								
Venous thrombosis	3	17/285	5.96%	15/282	5.32%	1.13 [0.59, 2.19]	56%	0.71
Catheter occlusion	3	18/350	5.14%	30/362	8.29%	0.64 [0.13, 3.10]	83%	0.58
Fibrin sleeve	1	5/134	3.73%	1/136	0.74%	5.07 [0.60, 42.86]	–	0.14
Port removal	3	12/369	3.25%	6/387	1.55%	2.05 [0.81, 5.21]	0%	0.13
Catheter Fracture	1	1/44	2.27%	0/39	0.00%	2.67 [0.11, 63.62]	–	0.54
Catheter misplacement	5	12/608	1.97%	25/622	4.02%	0.51 [0.27, 0.96]	35%	0.04
Inadvertent artery puncture	3	6/359	1.67%	8/375	2.13%	0.78 [0.28, 2.18]	57%	0.64
Port/Catheter-related bloodstream infection	4	8/484	1.65%	21/498	4.22%	0.37 [0.17, 0.81]	0%	0.01
Subcutaneous hematoma	2	2/160	1.25%	2/159	1.26%	0.99 [0.17, 5.66]	0%	0.99
Skin infection/necrosis around the port	3	3/342	0.88%	5/358	1.40%	0.63 [0.15, 2.62]	0%	0.53
Pneumothorax	5	0/608	0.00%	1/622	0.16%	0.36 [0.01, 8.83]	–	0.53
Infiltration/extravasation	1	0/134	0.00%	4/136	2.94%	0.11 [0.01, 2.07]	–	0.14

CI, Confidence interval; IJV, Internal Jugular Vein; RR, Risk ratio; SCV, Subclavian Vein.

### Complications assessed according to catheter days

According to catheter days, the SCV group showed higher rates of total complications (RR: 0.48 [0.35, 0.67], P < 0.0001, *I* ^2^ = 29%) and PRBIs (RR: 0.32 [0.14, 0.72], P = 0.006, *I* ^2^ = 0%). The rates of venous thrombosis, catheter occlusion, catheter fracture, fibrin sleeve, port removal, catheter misplacement, skin infection/necrosis around the port, inadvertent artery puncture, pneumothorax, and infiltration/extravasation were comparable between the two groups (**Table 3**, **Supplementary Figure S3**).

**Table 3:** Complications assessed according to catheter days.

Complications assessed according to catheter days	Studies involved	IJV		SCV		Risk ratio [95% CI]	*I^2^*	P-value
		Event/total	/1000 catheter days	Event/total	/1000 catheter days			
**Total**	4	58/299075	19.39%	99/256025	38.67%	0.48 [0.35, 0.67]	29%	< 0.0001
**Complications**								
Venous thrombosis	3	17/158183	10.75%	15/103745	14.46%	0.75 [0.37, 1.51]	9%	0.42
Catheter occlusion	3	18/183433	9.81%	30/189385	15.84%	0.60 [0.12, 3.15]	83%	0.55
Catheter Fracture	1	1/19536	5.12%	0/14742	0.00%	2.26 [0.09, 55.57]	–	0.62
Fibrin sleeve	1	5/115642	4.32%	1/66640	1.50%	2.88 [0.34, 24.66]	–	0.33
Port removal	2	11/256534	4.29%	6/218920	2.74%	1.83 [0.69, 4.87]	0%	0.23
Catheter misplacement	4	12/299075	4.01%	19/256025	7.42%	0.63 [0.31, 1.27]	26%	0.20
Port/Catheter-related bloodstream infection	4	8/299075	2.67%	21/256025	8.20%	0.32 [0.14, 0.72]	0%	0.006
Skin infection/necrosis around the port	2	3/163897	1.83%	5/174643	2.86%	0.62 [0.15, 2.60]	0%	0.52
Inadvertent artery puncture	1	1/140892	0.71%	5/152280	3.28%	0.22 [0.03, 1.85]	–	0.16
Pneumothorax	4	0/299075	0.00%	1/256025	0.39%	0.36 [0.01, 8.84]	–	0.53
Infiltration/extravasation	1	0/115642	0.00%	4/66640	6.00%	0.06 [0.00, 1.19]	–	0.07

CI, Confidence interval; IJV, Internal Jugular Vein; RR, Risk ratio; SCV, Subclavian Vein.

### Data of procedures

**Figure 4:**
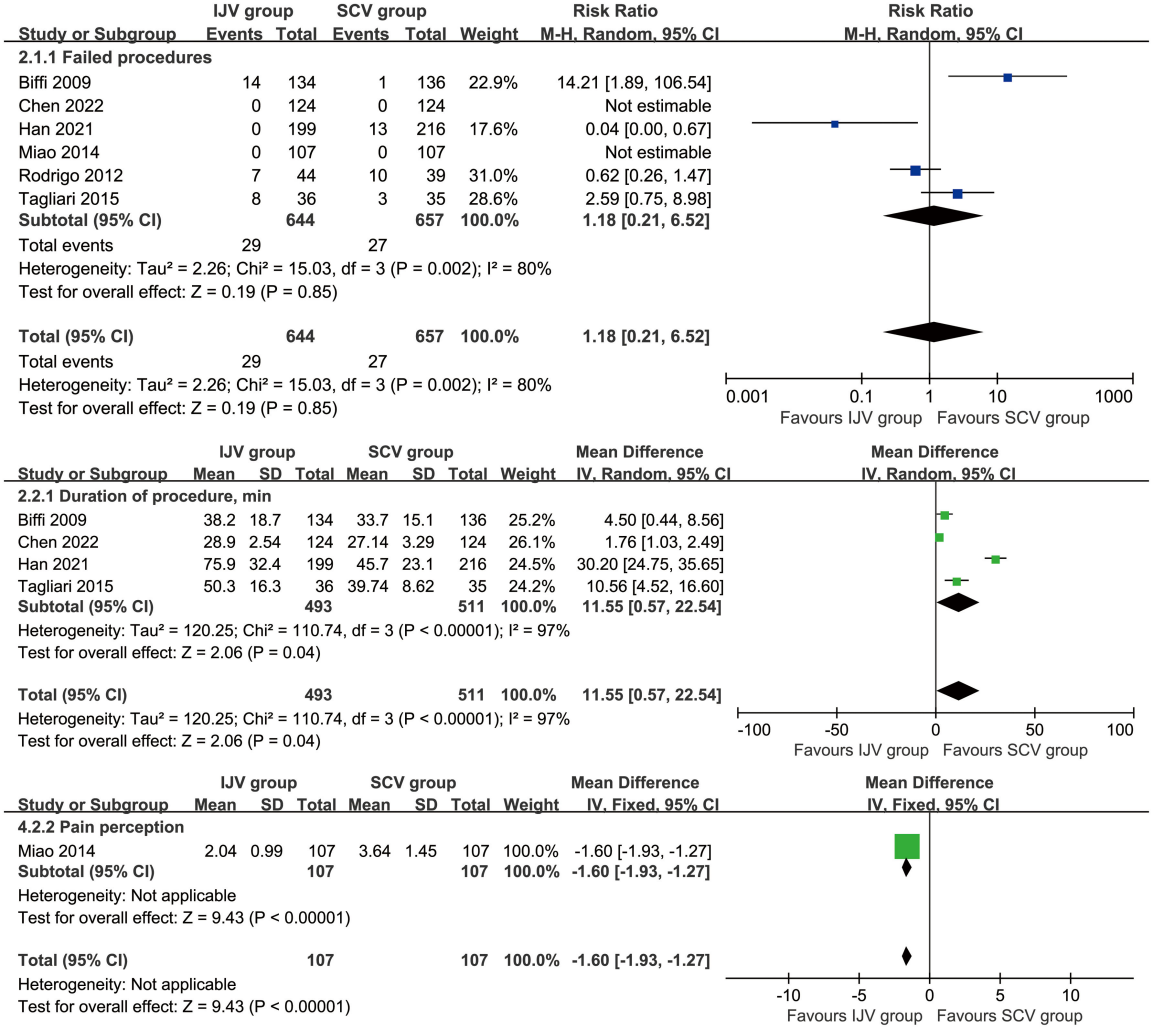
Forest plots of data of procedures associated with IJV versus SCV. The SCV group reported higher pain perception, shorter duration of the procedure, and similar procedural failures as compared with the IJV group.

The SCV group reported higher pain perception (MD: -1.60 [-1.93, -1.27], P < 0.00001), which indicating a perceptible and clinically meaningful increase in procedural pain. However, the duration of the procedure (MD: 11.55 [0.57, 22.54] minutes, P = 0.04, *I* ^2^ = 97%) was longer in the IJV group. The procedural failure rate was comparable between the two groups (**Figure 4**).

### Sensitivity analysis

The results for total complications assessed according to patients, duration of the procedure, and failed procedures, remained consistent after excluding individual studies in the sensitivity analysis (**Supplementary Figure S4**).

### Publication bias

**Figure 5:**
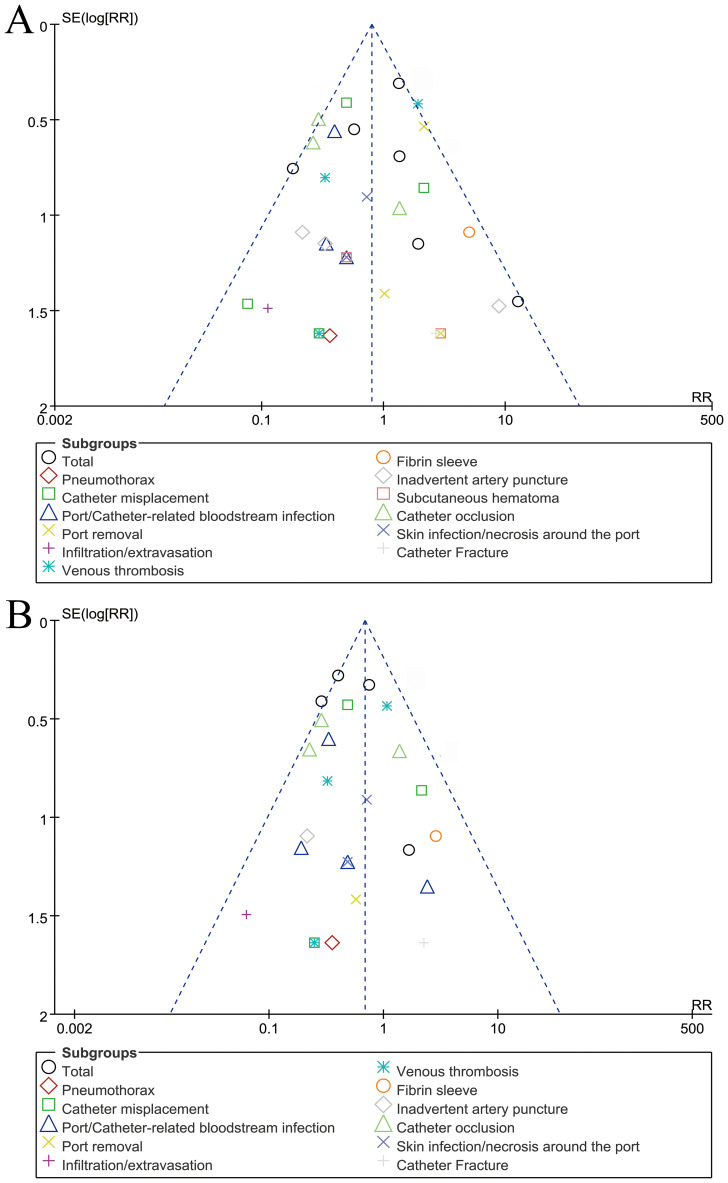
Funnel plots of complications assessed according to patients **(A)** or catheter days **(B)**. Visual inspection suggests symmetry, indicating a low risk of publication bias.

The funnel plot for complications assessed according to patients/catheter days, showed no significant publication bias (**Figure 5**).

## Discussion

Implantable ports for cancer chemotherapy play a critical role in patients undergoing long-term therapy (2). The choice of venous access site, whether the IJV or SCV, remains a topic of ongoing clinical debate, with no clear consensus on the optimal approach (23). Although previous studies have suggested differences in complications associated with these two approaches, no meta-analysis based on RCTs has comprehensively compared them to provide clear evidence for clinicians (16–22). Our meta-analysis suggests that IJV insertion is associated with fewer complications, including catheter misplacement and PRBIs, compared to SCV. However, IJV insertion is associated with a longer procedure duration, while pain perception is higher in the SCV group. These findings provide clinically relevant insights and contribute to the ongoing debate regarding the optimal central venous access site for cancer patients undergoing chemotherapy.

Complications related to central venous access play a crucial role in the decision-making process between the IJV and SCV for implantable port insertion. Our meta-analysis highlights significant differences in complications between the two venous access sites, with SCV being associated with a higher incidence of overall complications, catheter misplacement, and port/catheter-related bloodstream infections. Specifically, the total complication rate was lower in the IJV group, indicating that IJV insertion is relatively safer in terms of adverse events. Previous studies have reported similar findings, with a systematic review by Zhou et al. showing a lower risk of pneumothorax and catheter misplacement with IJV access (24). Moreover, several studies have pointed out that the IJV's more direct alignment with the superior vena cava reduces the chances of malposition and improves the overall success rate of the procedure (19). In contrast, while SCV insertion is associated with fewer complications related to catheter misplacement, it has been demonstrated to increase the likelihood of thrombosis and long-term dysfunction, as our results corroborate. Several studies have demonstrated that SCV access tends to lead to a higher risk of catheter occlusion, complicating chemotherapy administration (20, 21). Accurate tip positioning under ultrasound guidance has been shown to reduce both mechanical and infectious complications. Authors proposed an empirical ultrasonographic index for optimal tip placement in a surgical oncology setting, which could further enhance safety outcomes when used alongside site selection strategies (25). This can necessitate additional interventions, thus affecting long-term patient management. Therefore, clinicians must carefully weigh these potential risks when considering venous access routes.

Thrombosis and infection remain the most concerning complications of central venous access, as both can significantly impact the course of chemotherapy and the overall prognosis of cancer patients. Our study showed no substantial difference in thrombosis rates between the two groups. Velioğlu et al. reported a significant association between SCV access and thrombosis (26). The deeper anatomical location of the SCV and its proximity to the clavicle may contribute to the increased risk of catheter-induced thrombosis due to mechanical irritation and endothelial injury, a finding also corroborated by Ma et al. (27). Infection, particularly PRBIs, is a serious concern for patients with implantable ports. In our analysis, the SCV group showed a significantly higher incidence of PRBIs, supporting the findings of previous studies by Yanık et al., who noted that the deeper placement of catheters in the SCV increases the risk of bacterial colonization and infection due to longer catheter dwell times and reduced ability to perform routine maintenance (28). The higher infection rates in the SCV group underscore the importance of strict aseptic techniques during the insertion and management of central venous devices. Clinicians should carefully consider these risks when selecting the insertion site, particularly for patients with compromised immune systems undergoing cancer treatment.

Our study also assessed other procedural factors, including the duration of the procedure, the rate of failed procedures, and pain perception. The IJV group had a longer procedure duration, which aligned with previous research by Han et al., who reported that IJV insertion often requires more time due to its relatively more challenging anatomical positioning compared to the SCV (19). While the increased procedure time may be a disadvantage, it should be considered in the context of the overall complication profile, as the lower complication rate in the IJV group may justify the additional time required for insertion. Furthermore, the clinical impact of this prolonged duration deserves attention. Although the average extension of approximately 11.5 minutes may appear modest, it could contribute to increased patient discomfort during the procedure, especially in cases requiring multiple attempts or prolonged positioning. From a resource perspective, extended procedure times may affect operating room turnover, scheduling, and personnel workload in high-volume centers. Operator experience is also an important factor influencing procedure duration. As highlighted by Mey et al., ultrasound-guided IJV catheterization involves a learning curve, and increased familiarity with the technique can significantly reduce the time needed for successful cannulation (29). Therefore, the longer duration should be interpreted in light of institutional experience, patient tolerance, and overall clinical efficiency. Furthermore, pain perception associated with the procedure was notably higher in the SCV group. This could be attributed to the deeper anatomical location of the SCV, which may cause more discomfort during catheter placement (30). Pain management strategies should be carefully considered in clinical practice, particularly for patients undergoing repeated procedures. The study by Miao et al. further supports the finding that SCV access is associated with increased pain perception, likely due to the need for greater manipulation of the catheter during insertion (20).

Emerging technologies such as ECG-guided catheter placement, magnetic navigation tools, and ultrasound-based real-time tip confirmation are transforming the landscape of central venous access (31). These techniques have demonstrated high accuracy in tip positioning, thereby reducing complications such as catheter malposition, vascular trauma, and bloodstream infections (32). Their use also contributes to shorter procedure times and potentially fewer post-insertion adjustments (33). Incorporating these technologies into future clinical trials and meta-analyses will be crucial for refining evidence-based recommendations and achieving greater standardization across institutions. As these methods gain broader adoption, their comparative effectiveness and cost-efficiency should be critically evaluated to inform future updates of clinical practice guidelines.

There are several limitations that must be acknowledged. First, despite our systematic approach, heterogeneity in patient populations, surgical techniques, and postoperative care, as well as study design variability (e.g., lack of blinding, operator dependence, and follow-up duration differences), may affect the generalizability and reliability of our findings. These limitations highlight the need for higher-quality trials with standardized methodologies, including blinding, uniform operator training, and longer follow-up periods, to reduce bias and enhance evidence reliability. Second, although we focused on RCTs, which are considered the gold standard in clinical research, the sample sizes in some studies were relatively small, limiting the statistical power of our analyses. Future large-scale, multicenter trials are needed to confirm these findings and provide more reliable data on the safety and efficacy of IJV compared to SCV access. Third, due to the lack of stratified data and limited number of RCTs reporting complications, subgroup analyses by age, technique, or region could not be performed. Furthermore, long-term follow-up data were unavailable for most of the studies, limiting our ability to assess the long-term effects of these procedures.

## Conclusions

In summary, IJV access is associated with fewer complications, including catheter misplacement and PRBIs, compared to SCV. Although IJV insertion results in a longer procedure duration, it appears to be a less painful option for central venous access in cancer chemotherapy patients. Clinicians should carefully weigh the benefits and risks of each approach, considering patient-specific factors such as anatomy, the anticipated duration of chemotherapy, and the risk of complications. Further research with larger cohorts and longer follow-up is required to confirm these results and provide more robust clinical recommendations.

## Data Availability

The original contributions presented in the study are included in the article/**Supplementary Material**. Further inquiries can be directed to the corresponding author.

## Supplementary material

Supplementary Figure S1 Cochrane risk assessment. Each of the studies was considered to be of high quality.

Supplementary Figure S2 Forest plots of complications assessed according to patients associated with IJV versus SCV.

Supplementary Figure S3 Forest plots of complications assessed according to catheter days associated with IJV versus SCV.

Supplementary Figure S4 Sensitivity analysis. Total complications assessed according to patients **(A)**, duration of the procedure **(B)**, and failed procedures **(C)**. The results remain consistent after excluding individual studies in the sensitivity analysis.

Supplementary Table S1 Search strategy.

Supplementary Table S2 Methodological quality assessments (Jadad scale) of the included studies.

Supplementary Table S3 GRADE quality assessment by therapeutic strategy and study design for the outcomes.
