# Supplementary figures and images for: Comparing internal jugular vein and subclavian vein for central venous insertion of implantable ports in cancer chemotherapy: a meta-analysis of RCTs
Source: Front Oncol. 2025 May 26;15:1566757. doi: 10.3389/fonc.2025.1566757 (PMC12146329; doi:10.3389/fonc.2025.1566757)

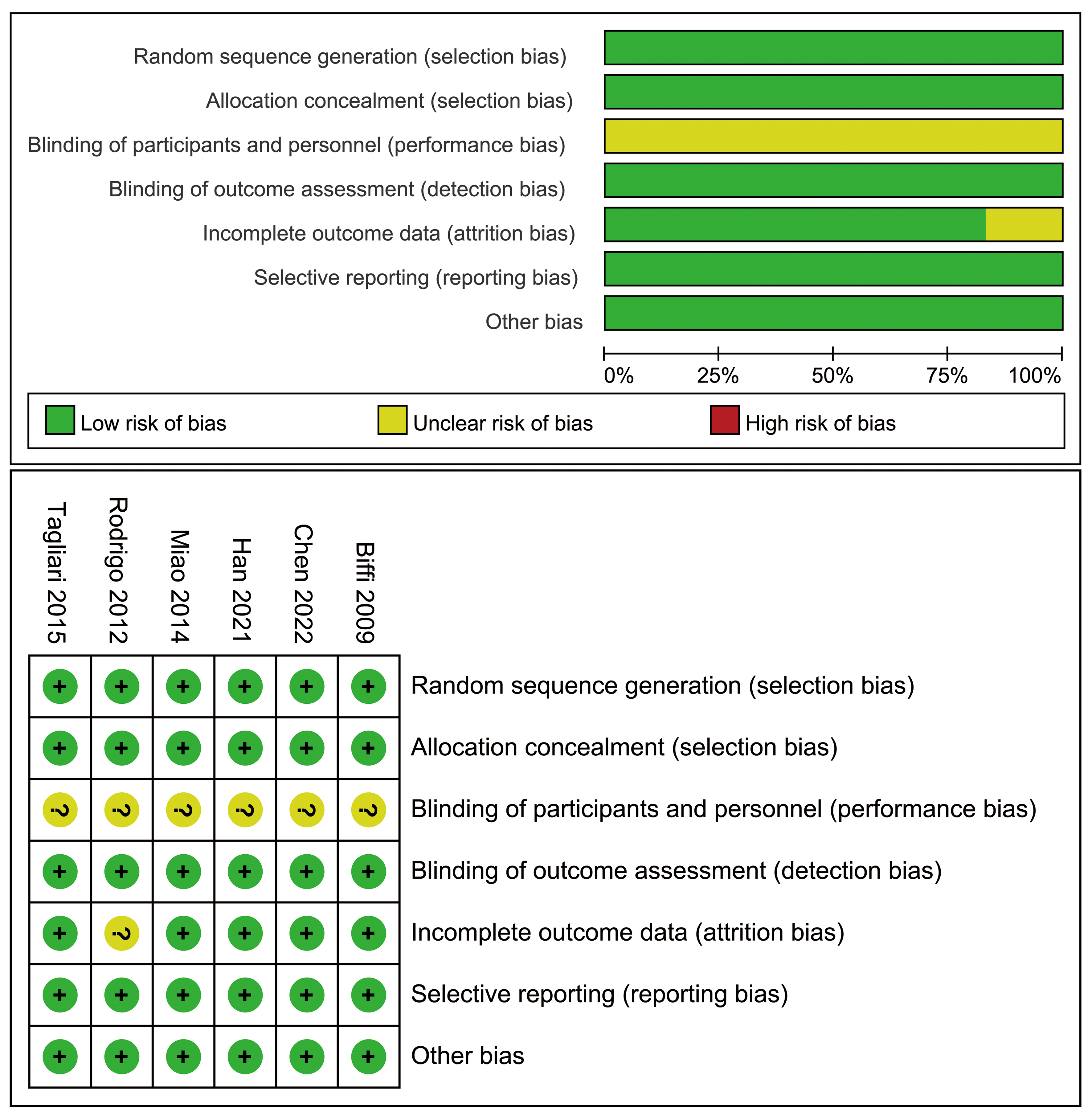

Supplement: Supplementary Figure S1 — Cochrane risk assessment. Each of the studies was considered to be of high quality. [file Image1.tif]

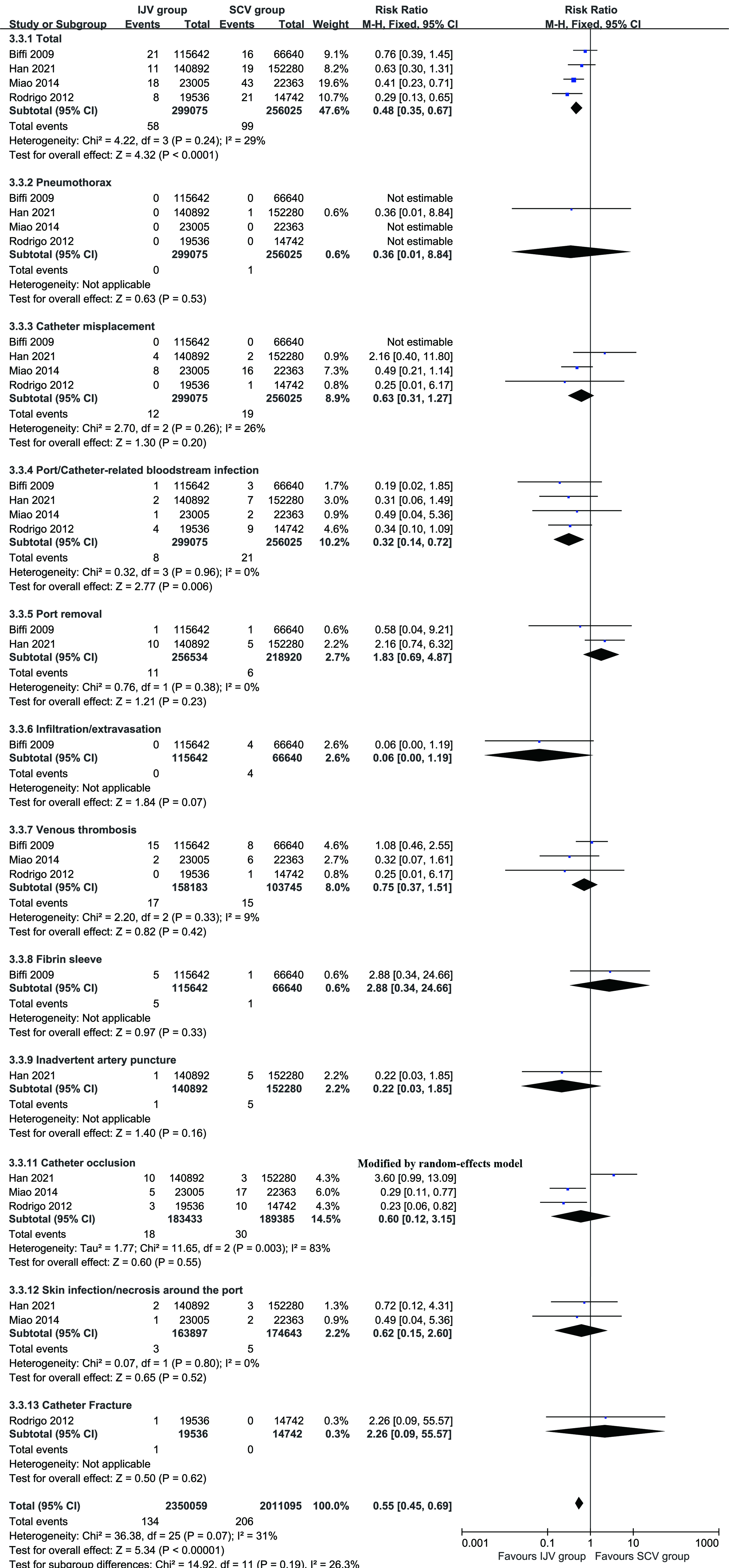

Supplement: Supplementary Figure S3 — Forest plots of complications assessed according to catheter days associated with IJV versus SCV. [file Image3.tif]

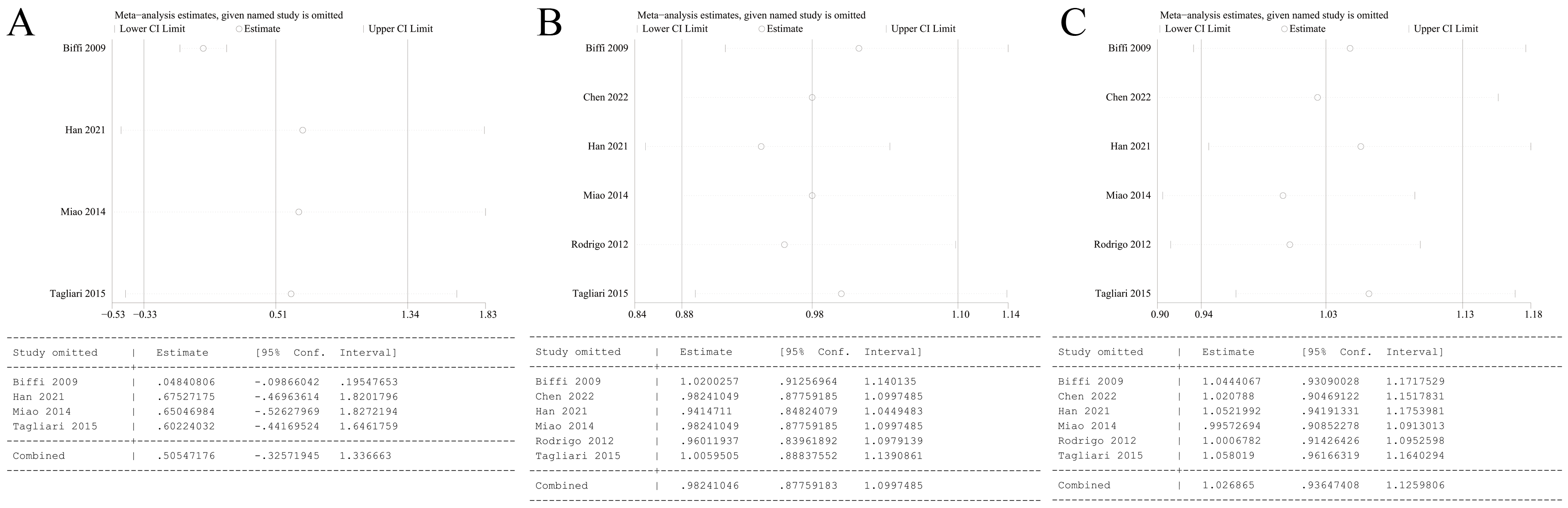

Supplement: Supplementary Figure S4 — Sensitivity analysis. Total complications assessed according to patients (A), duration of the procedure (B), and failed procedures (C). The results remain consistent after excluding individual studies in the sensitivity analysis. [file Image4.tif]
