# Supplementary material for: Comparing internal jugular vein and subclavian vein for central venous insertion of implantable ports in cancer chemotherapy: a meta-analysis of RCTs
Source: Front Oncol. 2025 May 26;15:1566757. doi: 10.3389/fonc.2025.1566757 (PMC12146329; doi:10.3389/fonc.2025.1566757)
Supplement: Supplementary Table S1 — Search strategy. [file Table1.docx]

**Table S1** Search strategy.

| **PubMed**  The database was searched on January 2, 2025, n=95.  Search Strategy:  **#1 Search: Subclavian vein[Title/Abstract] OR Subclavian[Title/Abstract] Sort by: Most Recent n = 20317**  **#2 Search: Internal jugular vein[Title/Abstract] OR Internal jugular[Title/Abstract] Sort by: Most Recent n = 9029**  **#3 Search: Randomized[Title/Abstract] OR Randomly[Title/Abstract] OR Randomised[Title/Abstract] n = 1221140**  **#1 and #2 and #3 n = 95** |
| --- |
| **Web of Science**  The database was searched on January 2, 2025, 2024, n=123.  Search Strategy:  Internal jugular vein OR Internal jugular (Abstract) AND Subclavian vein OR Subclavian (Abstract) AND Randomized OR Randomly OR Randomised (Abstract) and Preprint Citation Index (Exclude - Database) |
| **EMBASE**  The database was searched on January 2, 2025, 2024, n=214.  Search Strategy:  (Internal jugular vein:ti,ab,kw OR Internal jugular:ti,ab,kw:ti,ab,kw) AND (Subclavian vein:ti,ab,kw OR Subclavian:ti,ab,kw) AND **(Randomly**:ti,ab,kw **OR Randomised**:ti,ab,kw **OR Randomized**:ti,ab,kw**)** |
| **Cochrane Library**  The database was searched on January 2, 2025, 2024, n=32.  Search Strategy:  (Internal jugular vein OR Internal jugular**)** in Title Abstract Keyword AND (Subclavian vein OR Subclavian**)** in Title Abstract Keyword AND (**Randomized OR Randomly OR Randomised)** in Title Abstract Keyword - (Word variations have been searched) |
| **ScienceDirect**  The database was searched on January 2, 2025, n=433.  Search Strategy:  Title, abstract, keywords: ((“Internal jugular vein” OR “Internal jugular”) AND (“Subclavian vein” OR “Subclavian”) AND (“**Randomized**” **OR** “**Randomly**” **OR** “**Randomised**”)) |
| **Scopus**  The database was searched on January 2, 2025, n=237.  Search Strategy:  (TITLE-ABS-KEY (Internal jugular vein OR Internal jugular) AND TITLE-ABS-KEY (Subclavian vein OR Subclavian) AND TITLE-ABS-KEY (Randomized OR Randomly OR Randomised)) |

**Note:** 1. The combined text and medical subject heading (MeSH) terms used were: “**Internal jugular vein**”, “Subclavian vein”, and “**Randomized**”. **Internal jugular vein:** Internal jugular vein OR Internal jugular; Subclavian vein: Subclavian vein OR Subclavian; **Randomized:** Randomized OR Randomly OR Randomised.

2. Date ranges: From the inception up to January 2, 2025.

3. Language: Unrestricted language types.
