# Supplementary material for: Comparing internal jugular vein and subclavian vein for central venous insertion of implantable ports in cancer chemotherapy: a meta-analysis of RCTs
Source: Front Oncol. 2025 May 26;15:1566757. doi: 10.3389/fonc.2025.1566757 (PMC12146329; doi:10.3389/fonc.2025.1566757)
Supplement: Supplementary Table S2 — Methodological quality assessments (Jadad scale) of the included studies. [file Table2.docx]

**Table S2** Quality assessments of randomized clinical trials according to Jadad scale.

| **Study** | **Randomization** | **Concealment of allocation** | **Double blinding** | **Withdrawals and dropouts** | **Quality (score)** |
| --- | --- | --- | --- | --- | --- |
| Biffi 2009 [15], Biffi 2014 [16] | ** | ** |  | * | 6 |
| Chen 2022 [17] | ** | ** |  | * | 6 |
| Han 2021 [18] | ** | ** |  | * | 6 |
| Miao 2014 [19] | ** | ** |  |  | 5 |
| Rodrigo 2012 [20] | ** | ** |  |  | 5 |
| Tagliari 2015 [21] | ** | ** |  | * | 6 |
