# Supplementary material for: Comparing internal jugular vein and subclavian vein for central venous insertion of implantable ports in cancer chemotherapy: a meta-analysis of RCTs
Source: Front Oncol. 2025 May 26;15:1566757. doi: 10.3389/fonc.2025.1566757 (PMC12146329; doi:10.3389/fonc.2025.1566757)
Supplement: Supplementary Table S3 — GRADE quality assessment by therapeutic strategy and study design for the outcomes. [file Table3.docx]

**Table S3** GRADE quality assessment by therapeutic strategy and study design for the outcomes.

| **Primary outcomes** | **No. of Studies** | **No. of Participants** | | **Differences (95%CI) ^a^** | **Quality Assessment** | | | | | **Quality** |
| --- | --- | --- | --- | --- | --- | --- | --- | --- | --- | --- |
|  |  | **IJV** | **SCV** |  | **Risk of Bias^b^** | **Inconsistency** | **Indirectness** | **Imprecision** | **Publication Bias^c^** |  |
| **Characteristics** |  |  |  |  |  |  |  |  |  |  |
| Sex-Male | 5 | 187/600 | 202/618 | 0.98 [0.85, 1.12] | Low | No inconsistency | No indirectness | No imprecision | Unlikely | High |
| Port side-Left | 4 | 115/401 | 188/402 | 0.62 [0.28, 1.39] | Low | Serious (-1) | No indirectness | No imprecision | Unlikely | Medium |
| Age, year | 5 | 600 | 618 | 0.51 [-0.93, 1.95] | Low | Serious (-1) | No indirectness | No imprecision | Unlikely | Medium |
| Duration of implant, day | 4 | 484 | 498 | 114.06 [-109.51, 337.64] | Low | Serious (-1) | No indirectness | No imprecision | Unlikely | Medium |
| **Data of procedures** |  |  |  |  |  |  |  |  |  |  |
| Failed procedures | 6 | 29/644 | 27/657 | 1.18 [0.21, 6.52] | Low | Serious (-1) | No indirectness | No imprecision | Unlikely | Medium |
| Duration of procedure, min | 4 | 493 | 511 | 11.55 [0.57, 22.54] | Low | Serious (-1) | No indirectness | No imprecision | Unlikely | Medium |
| Pain perception | 1 | 107 | 107 | Pain perception | Low | No inconsistency | No indirectness | No imprecision | Unlikely | High |
| **Complications assessed according to patients** | | |  |  |  |  |  |  |  |  |
| Total | 5 | 60/608 | 110/622 | 0.52 [0.29, 0.93] | Low | Serious (-1) | No indirectness | No imprecision | Unlikely | Medium |
| Venous thrombosis | 3 | 17/285 | 15/282 | 1.13 [0.59, 2.19] | Low | No inconsistency | No indirectness | No imprecision | Unlikely | High |
| Catheter occlusion | 3 | 18/350 | 30/362 | 0.64 [0.13, 3.10] | Low | Serious (-1) | No indirectness | No imprecision | Unlikely | Medium |
| Fibrin sleeve | 1 | 5/134 | 1/136 | 5.07 [0.60, 42.86] | Low | No inconsistency | No indirectness | No imprecision | Unlikely | High |
| Port removal | 3 | 12/369 | 6/387 | 2.05 [0.81, 5.21] | Low | No inconsistency | No indirectness | No imprecision | Unlikely | High |
| Catheter fracture | 1 | 1/44 | 0/39 | 2.67 [0.11, 63.62] | Low | No inconsistency | No indirectness | No imprecision | Unlikely | High |
| Catheter misplacement | 5 | 12/608 | 25/622 | 0.51 [0.27, 0.96] | Low | No inconsistency | No indirectness | No imprecision | Unlikely | High |
| Inadvertent artery puncture | 3 | 6/359 | 8/375 | 0.78 [0.28, 2.18] | Low | No inconsistency | No indirectness | No imprecision | Unlikely | High |
| Port/Catheter-related bloodstream infection | 4 | 8/484 | 21/498 | 0.37 [0.17, 0.81] | Low | No inconsistency | No indirectness | No imprecision | Unlikely | High |
| Subcutaneous hematoma | 2 | 2/160 | 2/159 | 0.99 [0.17, 5.66] | Low | No inconsistency | No indirectness | No imprecision | Unlikely | High |
| Skin infection/necrosis around the port | 3 | 3/342 | 5/358 | 0.63 [0.15, 2.62] | Low | No inconsistency | No indirectness | No imprecision | Unlikely | High |
| Pneumothorax | 6 | 0/644 | 1/657 | 0.36 [0.01, 8.83] | Low | No inconsistency | No indirectness | No imprecision | Unlikely | High |
| Infiltration/extravasation | 1 | 0/134 | 4/136 | 0.11 [0.01, 2.07] | Low | No inconsistency | No indirectness | No imprecision | Unlikely | High |
| **Complications assessed according to catheter days** | | | |  |  |  |  |  |  |  |
| Total | 4 | 58/299075 | 99/256025 | 0.48 [0.35, 0.67] | Low | No inconsistency | No indirectness | No imprecision | Unlikely | High |
| Venous thrombosis | 3 | 17/158183 | 15/103745 | 0.75 [0.37, 1.51] | Low | No inconsistency | No indirectness | No imprecision | Unlikely | High |
| Catheter occlusion | 3 | 18/183433 | 30/189385 | 0.60 [0.12, 3.15] | Low | Serious (-1) | No indirectness | No imprecision | Unlikely | Medium |
| Catheter fracture | 1 | 1/19536 | 0/14742 | 2.26 [0.09, 55.57] | Low | No inconsistency | No indirectness | No imprecision | Unlikely | High |
| Fibrin sleeve | 1 | 5/115642 | 1/66640 | 2.88 [0.34, 24.66] | Low | No inconsistency | No indirectness | No imprecision | Unlikely | High |
| Port removal | 2 | 11/256534 | 6/218920 | 1.83 [0.69, 4.87] | Low | No inconsistency | No indirectness | No imprecision | Unlikely | High |
| Catheter misplacement | 4 | 12/299075 | 19/256025 | 0.63 [0.31, 1.27] | Low | No inconsistency | No indirectness | No imprecision | Unlikely | High |
| Port/Catheter-related bloodstream infection | 4 | 8/299075 | 21/256025 | 0.32 [0.14, 0.72] | Low | No inconsistency | No indirectness | No imprecision | Unlikely | High |
| Skin infection/necrosis around the port | 2 | 3/163897 | 5/174643 | 0.62 [0.15, 2.60] | Low | No inconsistency | No indirectness | No imprecision | Unlikely | High |
| Inadvertent artery puncture | 1 | 1/140892 | 5/152280 | 0.22 [0.03, 1.85] | Low | No inconsistency | No indirectness | No imprecision | Unlikely | High |
| Pneumothorax | 4 | 0/299075 | 1/256025 | 0.36 [0.01, 8.84] | Low | No inconsistency | No indirectness | No imprecision | Unlikely | High |
| Infiltration/extravasation | 1 | 0/115642 | 4/66640 | 0.06 [0.00, 1.19] | Low | No inconsistency | No indirectness | No imprecision | Unlikely | High |

**Abbreviations:** CI: Confidence interval; GRADE: Grading of Recommendations Assessment, Development and Evaluation; IJV: Internal Jugular Vein; MD: Mean difference; RCT: Randomized controlled trial; RR: Risk ratio; SCV: Subclavian Vein.

^a^ Differences: RR for dichotomous variables; MD for continuous variables.

^b^ Risk of bias assessed using the Jadad scale for RCTs.

^c^ Publication bias was explored through visual inspection of the funnel plots.
